# Urban-Rural Differences in Indoor and Outdoor Air Quality: A Comparative Study in Bangladesh

**DOI:** 10.3390/toxics13060509

**Published:** 2025-06-17

**Authors:** Masamitsu Kurata, Akira Hibiki, Kazushi Takahashi, Yutaka Matsumi

**Affiliations:** 1 Department of Economics, Sophia University, Tokyo 102-8554, Japan; 2 Graduate School of Economics and Management, Tohoku University, Sendai 980-8576, Japan; 3 Graduate School of Policy Studies, National Graduate Institute for Policy Studies (GRIPS), Tokyo 106-8677, Japan; 4 Institute for Space-Earth Environmental Research, Nagoya University, Nagoya 464-8601, Japan

**Keywords:** indoor air pollution, outdoor air pollution, solid fuel

## Abstract

Health hazards caused by indoor air pollution (IAP) remain a global concern, especially in developing countries. IAP has complex mechanisms related to outdoor air pollution (OAP) and various other factors, and their relationship needs to be clarified to examine effective policies. We conducted an indoor and outdoor air monitoring survey in urban, peri-urban, and rural areas in Bangladesh, one of the countries with the most severe air pollution. The results show that IAP is more severe in urban households than in rural households, with a five-fold difference in daily indoor ${PM}_{2.5}$ concentration between 117 $\mu g/m^{3}$ and 22 $\mu g/m^{3}$, respectively. Regression analysis reveals that IAP is strongly associated with OAP and is hardly affected by solid fuels used in well-ventilated outside kitchens. Our findings support the view that the mitigation of IAP in developing countries can be achieved not only through a transition to clean fuels, which often entails substantial costs, but also through more practical and accessible alternatives, such as the use of outdoor kitchens, electric fans, and careful management of behaviors such as indoor smoking and mosquito coil use.

## 1. Introduction

Health hazards caused by indoor air pollution (IAP) are a major global concern, as reported by various literature [1,2,3,4]. Ensuring good indoor air quality is critically important for public health, as people typically spend the majority of their time indoors [5,6]. Major sources of IAP include solid fuels such as firewood, coal, and animal dung [7]. Because solid fuels have been primarily used for cooking and heating in rural areas in the developing world, IAP is often regarded as a problem specific to those regions [8,9,10]. It is also common to emphasize the need for policies that encourage a shift from traditional solid fuels to cleaner fuels to reduce IAP [11].

However, at least two points should be noted when considering such views. First, the use of solid fuels does not always lead to severe IAP. Even when rural households use solid fuels, IAP can be mitigated through appropriate measures such as good ventilation and a separate kitchen [12]. Conversely, even when urban households use clean fuels, it can cause severe IAP due to unhealthy cooking methods [13]. Second, IAP is affected not only by intra-household activities but also by outdoor air pollution (OAP). If OAP is higher in urban areas than in rural areas, as is generally the case, IAP can also be higher in urban areas due to infiltration of outdoor pollutants [14,15]. However, the focus of research in urban areas tends to be biased toward OAP only, and studies targeting both OAP and IAP are still limited [16]. Therefore, it is essential to clarify the relationship between IAP, OAP, and solid fuel use to consider an effective policy for IAP.

This study focuses on Bangladesh, one of the countries with severe air pollution, and provides a comparative analysis of IAP among urban, peri-urban, and rural areas near the capital city of Dhaka. In Bangladesh, there is no consensus on whether IAP is more severe in rural or urban areas. For example, while Dasgupta et al. [12] indicated that IAP is worse in rural areas, Khalequzzaman et al. [17] reported the opposite. To add new findings, we conducted a 48-h monitoring survey for 50 households equipped with individual sensors for IAP and OAP. Combined with data on household characteristics and members’ behavior, we analyzed regional variations of IAP and associated factors.

The results show that IAP is much higher in urban and peri-urban areas than in rural areas. Regression analysis revealed that IAP is strongly associated with OAP and is hardly affected by solid fuels in well-ventilated outside kitchens away from living spaces, which is a common settlement structure in rural Bangladesh and other developing countries. We also confirmed the effectiveness of electric fans and the adverse effects of cigarette smoking and the use of mosquito coils.

## 2. Materials and Methods

### 2.1. Survey Area and Sampling of Households

**Figure 1:**
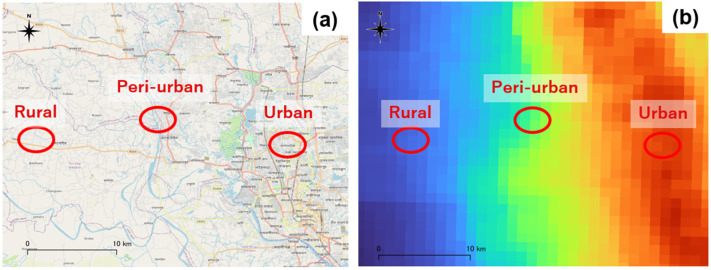
Survey area: (**a**) location of three areas in and nearby Dhaka, and (**b**) the level of ambient air pollution of nitrogen dioxides (${NO}_{2}$) measured using satellite (Sentinel-5P) in 2022 [19].

We conducted an air monitoring survey in Dhaka, the capital of Bangladesh, and in nearby peri-urban and rural areas in October 2022. These regions were selected because Dhaka is one of the most polluted cities in the world [18], and the surrounding area shows a large variation in air pollution within a small geographic zone. As shown in Figure 1, three study areas were selected to exploit the variation in OAP, namely, an urban area with high OAP, a peri-urban area with moderate OAP, and a rural area with low OAP. First, we identified Mirpur as a representative urban area, as it has a very high population density in Dhaka. Next, we selected Singair, the closest rural area to Mirpur, as a comparative site with similar climatic and geographical conditions. Finally, we selected Savar, located between the two areas, as the study site representing the peri-urban area.

In each area, we visited households as randomly as possible to find those willing to cooperate with the installation of sensors and face-to-face interviews. Finally, 50 households consented to the survey, including 10 in urban areas, 20 in peri-urban areas, and 20 in rural areas. The study protocol was approved by the Institutional Review Board of the Institute of Health Economics at the University of Dhaka.

### 2.2. Air Monitoring and Interview Survey

A palm-sized optical sensor (hereafter referred to as a “P-sensor”) was used to monitor ${PM}_{2.5}$ mass concentration [20]. The P-sensor is designed to estimate ${PM}_{2.5}$ based on the light scattering intensity distribution and is manufactured by Panasonic Corporation, a prominent Japanese multinational electronics company (https://www.panasonic.com/global/about.html, accessed on 11 June 2025), based on the verification of the high quality of the collected data [20]. According to previous studies using P-sensors [21,22,23], this study also estimated the mass concentration of ${PM}_{2.5}$ using a conversion factor of 1.4.

Two P-sensors were installed per household to quantify both IAP and OAP. One unit measuring IAP (indoor sensor) was placed in the living room or bedroom, and the other measuring OAP (outdoor sensor) was placed on the balcony or outside the front door. We installed the indoor sensor in the living room rather than the kitchen because kitchens in rural and peri-urban Bangladesh are often located separately outside the living space [12].

**Figure 2:**
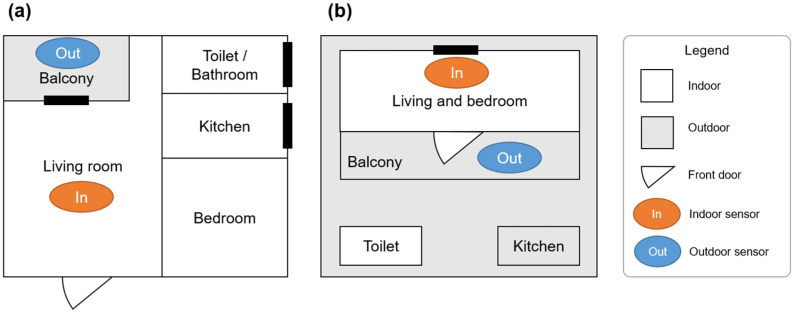
Typical house configurations in (**a**) urban and (**b**) rural areas.

Figure 2 shows typical house configurations in urban and rural areas and the locations where the two sensors are installed. In urban areas, most residences are in multi-story apartments equipped with balconies (Figure 2a), and the kitchen is generally located near the living room. The indoor sensor is installed in the living room, and the outdoor sensor is installed on the outdoor balcony. In rural and peri-urban areas, the main house is used as a living room and/or bedroom, with a toilet and a kitchen usually located outside (Figure 2b). The kitchen often has a roof but no walls on all sides, making it highly ventilated.

We measured IAP and OAP at 5-min intervals for approximately four days for each household. Due to the limited number of P-sensor equipment, monitoring was staggered by a few days in each area. Generally, sensors were installed in the afternoon of the first day of monitoring and were removed in the afternoon of the fourth day. Because the data from the first and last days contained noise due to the work required for installation and removal, only the data from the middle two full days (48 h) was used for the analysis. Depending on the purpose of the analysis, we aggregate the raw data at 5-min intervals into hourly or daily data.

In addition to air monitoring, interviews were conducted to collect data on various household characteristics. The data can be grouped into four main categories. The first is the type of fuel used for cooking, including solid fuels (such as firewood, agricultural residues, or animal dung), liquefied petroleum gas (LPG), natural gas, and electricity. The second concerns the structure of the house and kitchen, covering the size of the house, the number of rooms, whether the kitchen is located outside, and whether the kitchen is shared with other households. The third relates to household members’ behaviors that may affect IAP, such as daily cooking duration, smoking, use of mosquito coils, and use of electric fans or air conditioning. The fourth category includes other household characteristics, such as the gender and age of the household head and the household’s income level.

### 2.3. Data Analysis

We analyze regional differences in IAP and their determinants. The following regression analysis using hourly data are performed to examine the determinants:

$${IAP}_{ih}=\beta_{1}{Urban}_{i}+\beta_{2}{Periurban}_{i}+\beta_{3}{OAP}_{ih}+\beta_{4}{Solid}_{i}+\;\;\;\;\;\;\;\;\;\;\;\;\;\;\beta_{5}{OutsideKitchen}_{i}+\beta_{6}({Solid}_{i}\times {OutsideKitchen}_{i})+\sum_{k}\delta_{k}X_{ik}+\theta_{h}+\varepsilon_{ih}$$

where, ${IAP}_{ih}$ and ${OAP}_{ih}$ are the natural logarithms of indoor and outdoor air pollution measured in terms of ${PM}_{2.5}$, respectively, for household i and 48-h time h. $\beta_{3}$ indicates the partial correlation between IAP and OAP. ${Urban}_{i}$ and ${Periurban}_{i}$ are dummy variables for each area with rural as the reference group. The structural differences in IAP between rural and urban or peri-urban areas are reflected in $\beta_{1}$ or $\beta_{2}$.

${Solid}_{i}$ is a dummy variable indicating the use of solid fuels, such as firewood, agricultural residues, and animal dung. ${OutsideKitchen}_{i}$ is a dummy variable indicating that the household’s kitchen is located outside the living space. Since the impact of solid fuels on IAP would be mitigated if they are used in an outside kitchen, the interaction term is also added to the explanatory variables. In other words, while the effect of the solid fuels on IAP ($\beta_{4}$) would be positive in the case of the inside kitchen, its interaction with ${OutsideKitchen}_{i}$ ($\beta_{6}$) is expected to be negative.

$X_{ik}$ indicates other household characteristics (see the next section for details). Associations with various household-level characteristics are captured by $\delta_{k}$. Moreover, IAP may be subject to time-of-day factors that are common to all households, such as cooking time. $\theta_{h}$ indicates the 24-h time fixed effects controlling these factors. $\varepsilon_{ih}$ is a normal error term. Standard errors are clustered at the household level.

## 3. Results

### 3.1. Characteristics of Sample Households

The characteristics of the sample households are summarized in Table 1. For cooking fuels, many households use a combination of different types of fuels. In rural areas, most households use solid fuels, while half also use LPG. In peri-urban areas, all households use natural gas, although some also use solid fuel and/or LPG. Only clean fuels are used in urban areas: eight households use natural gas, and four use LPG.

The location of kitchens differed among areas, with 100% of rural areas having outdoor kitchens, 80% in peri-urban areas, and 10% in urban areas. Sharing kitchens with other households is particularly common in peri-urban areas (85%). The cooking time per day was approximately 2.5 to 3 h, with no significant differences among regions.

In the context of Bangladesh, other possible causes of IAP besides cooking are cigarette smoking and the use of mosquito coils. The percentage of smoking was highest among peri-urban households (40%). Mosquito coils are widely used in all areas to prevent infectious diseases such as dengue fever. More than 85% of rural and peri-urban areas use them regularly in their homes.

For cooling, almost all households use fans. Air conditioner use is limited to 10%, even in urban areas. As for housing, the area is larger in rural households, but the number of rooms is similar in all areas. The other notable difference between areas is household income, which is 1.8 times higher in urban areas than in rural areas.

### 3.2. Representative Cases by Region

**Figure 3:**
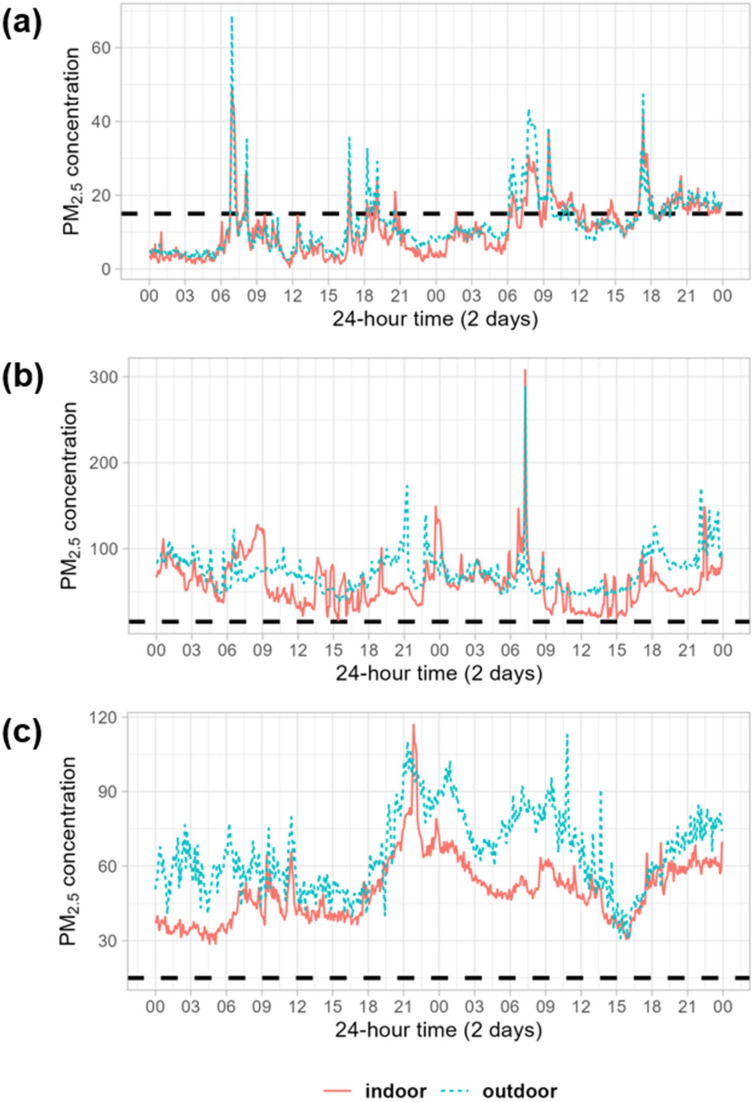
Representative cases of indoor and outdoor ${PM}_{2.5}$ concentrations ($\mu g/m^{3}$) in (**a**) rural, (**b**) peri-urban, and (**c**) urban areas. The horizontal dashed line indicates 15 $\mu g/m^{3}$, the air quality guideline level of WHO [24].

For illustrative purposes, Figure 3 shows the time paths of ${PM}_{2.5}$ concentrations in a representative household in each area. The WHO set the air quality guideline level for 24 h at 15 $\mu g/m^{3}$, which is indicated by the horizontal dashed line in the figure. For the rural household (Figure 3a), IAP generally meets the guideline level despite the use of solid fuels, except for peaks at breakfast and dinner preparation times. However, IAP exceeded the guideline level in the peri-urban and urban regions (Figure 3b,c). This peri-urban household uses natural gas and firewood, while the urban household uses only natural gas. The three cases also showed a clear positive correlation between IAP and OAP. In the rural and peri-urban cases, both indoor and outdoor ${PM}_{2.5}$ moved in tandem at similar levels. The trend is similar for urban households, but the level is higher for OAP.

### 3.3. IAP and OAP Variation by Region

**Figure 4:**
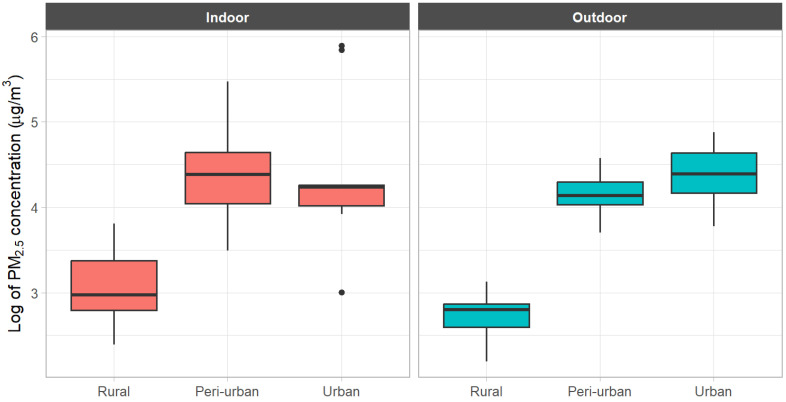
Comparison of indoor and outdoor ${PM}_{2.5}$ concentration ($\mu g/m^{3}$) by region.

Table 2 shows the indoor and outdoor ${PM}_{2.5}$ concentrations by region using daily and hourly data. For most of the values for IAP and OAP, rural areas had lower levels than urban areas. The mean ${PM}_{2.5}$ concentration for urban indoor and outdoor environments was about five times higher than that for rural areas. Even at the median, both IAP and OAP were much lower in rural areas than in urban and peri-urban areas. Figure 4 confirms this relationship on a logarithmic scale.

**Figure 5:**
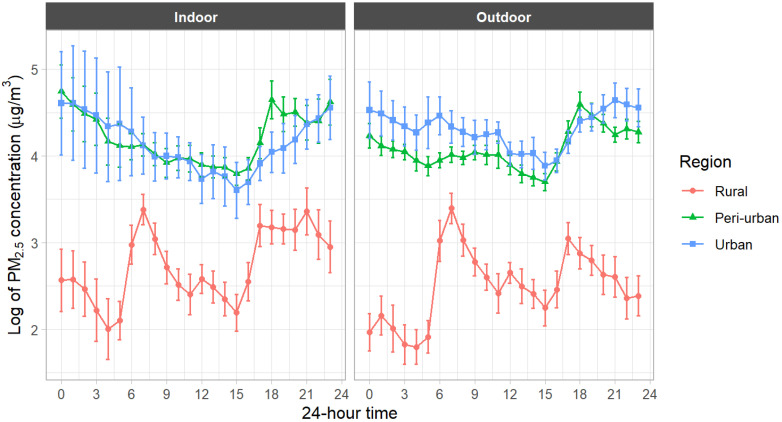
${PM}_{2.5}$ concentration by the time of day. The bar indicates a 95% confidence interval.

Table 3 shows the variation in indoor and outdoor ${PM}_{2.5}$ concentrations over a day based on hourly data. Rural IAP and OAP are lower than those for urban and peri-urban areas at all times of the day. The observation is also confirmed by Figure 5, which compares the hourly means on a logarithmic scale.

It should be noted that nighttime IAP (0:00−6:00 and 18:00−24:00) is relatively high in urban and peri-urban areas. There are two possible reasons. First, the daytime natural gas supply was restricted in Dhaka and its suburbs during this survey in October 2022. During the interviews, urban and peri-urban residents reported that they cooked intensively late at night and early in the morning due to the gas crisis caused by high international energy prices. Second, as Table 2 confirms, OAP generally tends to be higher in the evening. This result is consistent with previous studies and may include the influence of the evening rush hour [15,25]. This higher outdoor ${PM}_{2.5}$ may have eventually contributed to IAP in urban and peri-urban areas.

### 3.4. Determinants of IAP

**Figure 6:**
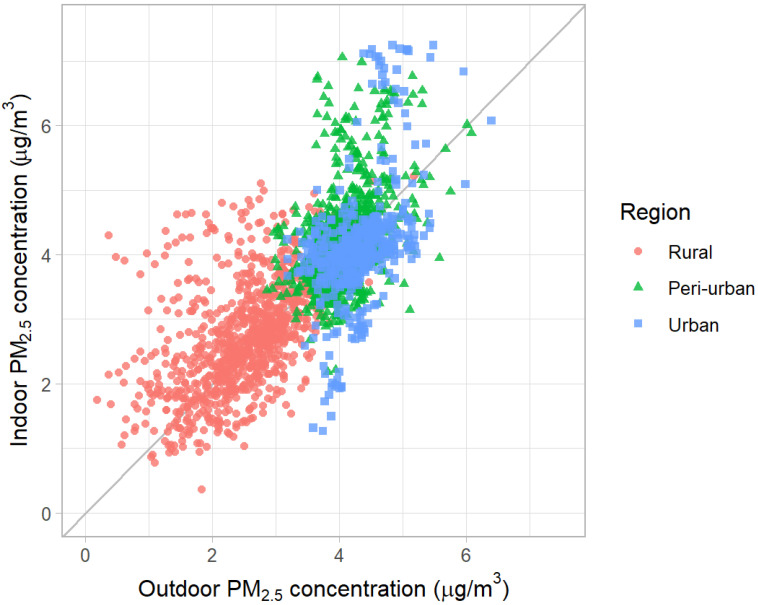
Correlation between indoor and outdoor ${PM}_{2.5}$ concentration.

The above analysis shows that urban households using only clean fuels are exposed to higher IAP than rural households using mainly solid fuels. Apart from cooking fuels, what factors contribute to this difference? Because we selected three study areas exploiting differences in OAP (Figure 1), OAP could be an important factor. Figure 6 shows the strong correlation between IAP and OAP on a logarithmic scale (R = 0.73).

Table 4 shows the regression analysis. Column 1 uses only urban and peri-urban dummies as explanatory variables, with rural households as the reference group. The result again confirms the higher IAP in urban and peri-urban areas. However, the coefficients of regional dummies become much smaller when OAP is added as an explanatory variable in Column 2. This means that most of the regional differences in IAP can be explained by OAP. The coefficient of OAP indicates that a 1 percent increase in OAP is associated with a 0.57 percent increase in IAP.

In Column 3, we added all explanatory variables described in Table 1. The results are summarized in the following three major findings. First, the coefficients for the solid fuels and the outside kitchen are both positive, but their interaction is negative, as expected. This indicates that although solid fuels are an increasing factor in IAP, their impact is significantly reduced when the kitchen is located outside the living space. Second, other factors increasing IAP are cigarette smoking and the use of mosquito coils. Third, the use of fans and larger house sizes contribute to the decrease in IAP. Most other variables omitted from the table were not statistically significant.

## 4. Discussion

The results indicate that both IAP and OAP are higher in urban and peri-urban areas than in rural areas. This finding is consistent with Khalequzzaman et al. [17], who compare kitchen IAP and OAP between rural and urban households in Dhaka. However, a key difference between their study and ours is that all urban households in their sample use solid fuels (firewood). In our study, by contrast, most urban and peri-urban households use clean fuels such as LPG and natural gas. Despite this, indoor air quality remains worse than in rural areas. As noted earlier, this may be partly due to limited daytime gas supply during the survey period, which leads to intensive cooking activities at night and in the early morning. Even if this is the case, our findings suggest that the use of clean fuels alone may not be sufficient to achieve low IAP.

Another relevant comparison is with Dasgupta et al. [12], who report that rural households are exposed to higher IAP than urban households. Two main possible reasons explain the divergence from our results. First, their sample includes households with a wide variety of kitchen arrangements. For example, households that use solid fuels and have kitchens located inside the main dwelling exhibit higher IAP. In contrast, all rural households in our sample use well-ventilated outside kitchens. This sampling characteristic may have led to an underestimation of rural IAP in our study. Nevertheless, their finding that IAP is substantially reduced when the kitchen is detached or located outdoors is consistent with our results. This observation is particularly relevant, given that outdoor kitchens are widely used not only in Bangladesh but also in many other developing countries [26].

Second, while the study by Dasgupta et al. [12] was conducted in 2003–2004, OAP in Dhaka has been steadily deteriorating since then. The city’s average annual ${PM}_{2.5}$ concentration increased from approximately 50 $\mu g/m^{3}$ in 2001 to around 70 $\mu g/m^{3}$ in 2019 [27]. In Mirpur, our urban study site, it reached as high as 80 $\mu g/m^{3}$. This worsening OAP has substantially contributed to higher IAP in urban areas, accounting for the different results between their study and ours conducted in 2022.

We acknowledge several limitations in this study. First, it focuses solely on ${PM}_{2.5}$ and does not include other pollutants such as O_3_ or NO_x_, which may behave differently under similar conditions. Second, the sample size is relatively small (50 households, including only 10 from urban areas), and the monitoring duration is short-term, limited to 48 h in October 2022. In addition, air quality was measured only in outdoor areas and living rooms, excluding potentially relevant spaces such as kitchens. These factors may introduce biases related to the seasonality of air pollution and household characteristics. Finally, the regression analysis does not control for meteorological conditions. Although the three study sites are geographically close and likely share similar temperatures, other environmental variables such as wind speed and direction may vary by household location.

Beyond these limitations, several issues remain for future research. Our findings confirmed that smoking and the use of mosquito coils contribute to elevated IAP. To better understand the extent to which these effects vary with the frequency and timing of such behaviors, more detailed behavioral data are required. Furthermore, although all rural households in this study had outside kitchens, emissions from these kitchens may affect OAP more than IAP. However, since the outdoor sensors were installed on the balcony of the main dwelling, we were unable to directly capture emissions from the kitchens. Future studies should consider deploying sensors placed both inside and around kitchen areas to more accurately assess their contributions to OAP.

## 5. Conclusions

This study examined the status and determinants of IAP in Bangladesh, a country known for experiencing severe air pollution. Taking advantage of the substantial variation in ambient air pollution in and around the capital city of Dhaka, the concentrations of ${PM}_{2.5}$ were measured both indoors and outdoors across 50 households located in three distinct regions: urban, peri-urban, and rural. By combining data on various household characteristics with hourly data on ${PM}_{2.5}$, the determinants for IAP were examined using regression analysis.

The results indicate that IAP is more pronounced in urban and peri-urban areas than in rural areas. In rural households, the daily concentration of ${PM}_{2.5}$ in living rooms remained relatively low, averaging $22\;\mu g/m^{3}$ per day, despite the use of solid fuels. In contrast, urban and peri-urban households recorded substantially higher daily ${PM}_{2.5}$ concentrations, approximately $100\;\mu g/m^{3}$, even though clean energy sources such as LPG and natural gas were predominantly used. This result is primarily explained by the infiltration of outdoor air pollutants, which is particularly severe in urban settings. In addition to OAP, smoking and the use of mosquito coils are also associated with high IAP.

The mechanisms underlying IAP are multifaceted, reflecting complex interaction with OAP and various household characteristics. Our findings suggest that high levels of OAP can offset the benefits of clean fuel use in urban areas, thereby limiting the effectiveness of fuel-switching policies alone. To mitigate IAP in developing-country contexts, a comprehensive approach may be needed—one that includes not only transitioning to clean fuels but also practical interventions such as promoting well-ventilated outdoor kitchens, using electric fans to enhance airflow, and encouraging behavioral changes such as avoiding indoor smoking and reducing mosquito coil use. Of course, this comprehensive approach should also include continued efforts to reduce reliance on solid fuels, which remain major contributors to IAP, particularly in poorly ventilated indoor environments.

## Figures and Tables

**Table 1:** Characteristics of sample households.

	Rural (N = 20)	Peri-Urban (N = 20)	Urban (N = 10)
Cooking fuel: solid fuels	19 (95%)	9 (45%)	0 (0%)
Cooking fuel: LPG	11 (55%)	6 (30%)	4 (40%)
Cooking fuel: natural gas	0 (0%)	20 (100%)	8 (80%)
Cooking fuel: electricity	1 (5%)	2 (10%)	1 (10%)
Outside kitchen	20 (100%)	16 (80%)	1 (10%)
Sharing kitchen with other households	1 (5%)	17 (85%)	4 (40%)
Smoking by any household members	4 (20%)	8 (40%)	1 (10%)
Use of mosquito coils	17 (85%)	18 (90%)	6 (60%)
Use of a fan	20 (100%)	19 (95%)	10 (100%)
Use of an air conditioner	0 (0%)	1 (5%)	1 (10%)
Female head of household	2 (10%)	3 (15%)	1 (10%)
Age of the head of household	53.8	38.1	47.9
Number of household members (person)	3.7	3.4	4.1
Size of housing (decimal)	16.4	5.0	1.9
Number of rooms	3.6	3.0	4.3
Average cooking time per day (hour)	2.9	2.5	2.5
Average monthly household income (Taka)	25,950	27,750	47,200

Note: “Cooking fuel: solid fuels” is a dummy variable that indicates the use of firewood, agricultural residues, or animal dung. “Decimal” is a unit of area commonly used in Bangladesh (1 decimal = 40.5 m^2^).

**Table 2:** Indoor and outdoor ${PM}_{2.5}$ concentration ($\mu g/m^{3}$).

		Daily Mean ${PM}_{2.5}$ Concentration			Hourly Mean ${PM}_{2.5}$ Concentration		
		Rural	Peri-Urban	Urban	Rural	Peri-Urban	Urban
Indoor	Mean	22.2	95.0	117.0	22.2	95.0	117.0
	Standard deviation	10.2	55.9	124.3	23.2	122.3	229.9
	Minimum	7.2	32.5	18.7	0.1	8.9	3.5
	Median	21.6	76.1	66.5	14.7	62.0	58.9
	Maximum	47.1	260.9	423.5	185.0	1162.8	1398.6
Outdoor	Mean	15.8	64.9	83.6	15.8	64.9	83.6
	Standard deviation	5.3	15.3	27.4	13.0	34.5	48.9
	Minimum	5.2	36.6	40.4	0.7	17.4	24.1
	Median	15.8	63.8	78.8	13.1	58.1	73.7
	Maximum	28.6	124.3	141.2	175.7	436.7	598.9
Num. of observation		40	40	20	960	960	480

**Table 3:** Mean ${PM}_{2.5}$ concentration ($\mu g/m^{3}$) by the time of day.

	Time of Day	Rural	Peri-Urban	Urban
Indoor	0:00–6:00	18.3	142.3	252.8
	6:00–12:00	21.7	61.7	74.0
	12:00–18:00	17.1	55.4	49.0
	18:00–24:00	31.7	120.4	92.1
Outdoor	0:00–6:00	8.8	61.5	96.3
	6:00–12:00	22.0	58.0	79.6
	12:00–18:00	15.5	53.0	58.4
	18:00–24:00	16.8	86.9	100.2
Num. of observation		960	960	480

**Table 4:** Regression results: determinants of indoor air pollution (${PM}_{2.5}$).

	(1)	(2)	(3)
Urban	1.101 ***	0.265	0.191
	(0.233)	(0.232)	(0.317)
Peri-urban	1.726 ***	0.696 **	0.052
	(0.125)	(0.207)	(0.310)
Log(Outdoor air pollution)		0.565 ***	0.571 ***
		(0.103)	(0.095)
Solid fuel			0.360 *
			(0.173)
Outside kitchen			0.503 *
			(0.189)
Solid fuel × outside kitchen			−0.509 *
			(0.236)
Smoking			0.396*
			(0.179)
Mosquito coil			0.339 *
			(0.156)
Fan			−1.011 **
			(0.368)
House size			−0.030 *
			(0.015)
Controlling for other household characteristics	No	No	Yes
24-h time fixed effects	Yes	Yes	Yes
Num. of observation	2400	2390	2390
Adjusted R2	0.523	0.587	0.637

Notes: Hourly data were used in the regression analysis. The explained variable is the logarithmic scale of indoor ${PM}_{2.5}$ concentration ($\mu g/m^{3}$). Standard errors, clustered at the household level, are shown in parentheses. Significance levels: * *p* < 0.05, ** *p* < 0.01, *** *p* < 0.001.

## Data Availability

The data presented in this study are available on request from the corresponding author.

## Abbreviations

The following abbreviations are used in this manuscript:

IAP	Indoor air pollution
OAP	Outdoor air pollution
${PM}_{2.5}$	Particulate matter (diameter ≤ 2.5 μm)

## References

[1] Van Tran V., Park D., Lee Y.C. (2020). Indoor air pollution related human diseases recent trends in the control improvement of indoor air quality. Int. J. Environ. Res. Publ. Health.

[2] Pratiti R., Vadala D., Kalynych Z., Sud P. (2020). Health effects of household air pollution related to biomass cook stoves in resource limited countries and its mitigation by improved cookstoves. Environ. Res..

[3] Dominski F.H., Branco J.H.L., Buonanno G., Stabile L., da Silva M.G., Andrade A. (2021). Effects of air pollution on health: A mapping review of systematic reviews and meta-analyses. Environ. Res..

[4] Pillarisetti A., Ye W., Chowdhury S. (2022). Indoor air pollution and health: Bridging perspectives from developing and developed countries. Annu. Rev. Environ. Resour..

[5] Klepeis N.E., Nelson W.C., Ott W.R., Robinson J.P., Tsang A.M., Switzer P., Behar J.V., Hern S.C., Engelmann W.H. (2001). The National Human Activity Pattern Survey (NHAPS): A resource for assessing exposure to environmental pollutants. J. Expo. Sci. Environ. Epidemiol..

[6] Yun H., Seo J.H., Kim Y.G., Yang J. (2025). Impact of scented candle use on indoor air quality and airborne microbiome. Sci. Rep..

[7] Chen B.H., Hong C.J., Pandey M.R., Smith K.R. (1990). Indoor air pollution in developing countries. World Health Stat. Q..

[8] Smith K.R. (2002). Indoor air pollution in developing countries: Recommendations for research. Indoor Air..

[9] Shen G. (2017). Mutagenicity of particle emissions from solid fuel cookstoves: A literature review and research perspective. Environ. Res..

[10] Arku R.E., Ezzati M., Baumgartner J., Fink G., Zhou B., Hystad P., Brauer M. (2018). Elevated blood pressure and household solid fuel use in premenopausal women: Analysis of 12 Demographic and Health Surveys (DHS) from 10 countries. Environ. Res..

[11] Amegah A.K., Jaakkola J.J. (2016). Household air pollution and sustainable development goals. Bull. World Health Organ..

[12] Dasgupta S., Huq M., Khaliquzzaman M., Pandey K., Wheeler D. (2006). Indoor air quality for poor families: New evidence from Bangladesh. Indoor Air..

[13] Kang K., Kim H., Kim D.D., Lee Y.G., Kim T. (2019). Characteristics of cooking-generated PM_10_ and PM_2.5_ in residential buildings with different cooking and ventilation types. Sci. Total Environ..

[14] Shupler M., Godwin W., Frostad J., Gustafson P., Arku R.E., Brauer M. (2018). Global estimation of exposure to fine particulate matter (PM_2.5_) from household air pollution. Environ. Int..

[15] Nahian S., Roy S., Choudhury T.R., Begum B.A., Salam A. (2024). Indoor particulate matter exposure and correlation of PM_2.5_ with lung efficacy and SpO_2_ level of Dhaka City Dwellers. Air Qual. Atmos. Health.

[16] Dyer G.M., Khomenko S., Adlakha D., Anenberg S., Behnisch M., Boeing G., Esperon-Rodriguez M., Gasparrini A., Khreis H., Kondo M.C. (2024). Exploring the nexus of urban form, transport, environment and health in large-scale urban studies: A state-of-the-art scoping review. Environ. Res..

[17] Khalequzzaman M., Kamijima M., Sakai K., Ebara T., Hoque B.A., Nakajima T. (2011). Indoor air pollution and health of children in biomass fuel-using households of Bangladesh: Comparison between urban and rural areas. Environ. Health Prev. Med..

[18] IQAir (2023). World Air Quality Report 2023: Region and City PM2.5 Ranking. https://www.iqair.com/dl/2023_World_Air_Quality_Report.pdf.

[19] European Space Agency (2021). Copernicus Sentinel-5P (Processed by ESA), TROPOMI Level 2 Nitrogen Dioxide Total Column Products. Version 02. https://sentinels.copernicus.eu/web/sentinel/data-products/-/asset_publisher/fp37fc19FN8F/content/sentinel-5-precursor-level-2-nitrogen-dioxide.

[20] Nakayama T., Matsumi Y., Kawahito K., Watabe Y. (2018). Development and evaluation of a palm-sized optical PM_2.5_ sensor. Aerosol Sci. Technol..

[21] Dhaka S.K., Chetna V., Kumar V., Panwar V., Dimri A.P., Singh N., Patra P.K., Matsumi Y., Takigawa M., Nakayama T. (2020). PM_2.5_ diminution and haze events over Delhi during the COVID-19 lockdown period: An interplay between the baseline pollution and meteorology. Sci. Rep..

[22] Ly B.T., Matsumi Y., Nakayama T., Sakamoto Y., Kajii Y., Nghiem T.D. (2018). Characterizing PM_2.5_ in Hanoi with new high temporal resolution sensor. Aerosol Air Qual. Res..

[23] Othman M., Latif M.T., Yee C.Z., Norshariffudin L.K., Azhari A., Halim N.D.A., Alias A., Sofwan N.M., Hamid H.H.A., Matsumi Y. (2020). PM_2.5_ and ozone in office environments and their potential impact on human health. Ecotoxicol. Environ. Saf..

[24] World Health Organization (2021). WHO global air quality guidelines: Particulate matter (PM2.5 and PM10). Ozone, Nitrogen Dioxide, Sulfur Dioxide and Carbon Monoxide. World Health Organization. https://apps.who.int/iris/handle/10665/345329.

[25] Mahmood A., Hu Y., Nasreen S., Hopke P.K. (2019). Airborne particulate pollution measured in Bangladesh from 2014 to 2017. Aerosol Air Qual. Res..

[26] Langbein J., Peters J., Vance C. (2017). Outdoor cooking prevalence in developing countries and its implication for clean cooking policies. Environ. Res. Lett..

[27] Rahman M., Meng L. (2024). Examining the Spatial and Temporal Variation of PM_2.5_ and Its Linkage with Meteorological Conditions in Dhaka, Bangladesh. Atmosphere.

